# Tumor *Akkermansia muciniphila* predicts clinical response to immune checkpoint inhibitors in non-small-cell lung cancer patients with low PD-L1 expression

**DOI:** 10.3389/fimmu.2025.1528594

**Published:** 2025-09-08

**Authors:** Takashi Shimizu, Ryotaro Ohkuma, Mayumi Homma, Shingo Nakayama, Yosuke Sasaki, Satoshi Muto, Katsuaki Ieguchi, Makoto Watanabe, Akashi Taguchi, Daisuke Takayanagi, Youichiro Wada, Atsushi Horiike, Yutaro Kubota, Hirotsugu Ariizumi, Masahiro Shimokawa, Yuya Hirasawa, Tomoyuki Ishiguro, Risako Suzuki, Nana Iriguchi, Emiko Mura, Kiyoshi Yoshimura, Mayumi Tsuji, Yuji Kiuchi, Hiroyuki Suzuki, Toshiko Yamochi

**Affiliations:** 1 Department of Clinical Diagnostic Oncology, Clinical Research Institute for Clinical Pharmacology and Therapeutics, Showa Medical University, Tokyo, Japan; 2 Clinical Research Institute for Clinical Pharmacology and Therapeutics, Showa Medical University, Tokyo, Japan; 3 Division of Medical Oncology, Department of Medicine, School of Medicine, Showa Medical University, Tokyo, Japan; 4 Department of Pathology, Showa Medical University School of Medicine, Tokyo, Japan; 5 Department of Chest Surgery, School of Medicine, Fukushima Medical University, Fukushima, Japan; 6 Department of Pharmacology, School of Medicine, Showa Medical University, Tokyo, Japan; 7 Pharmacological Research Center, Showa Medical University, Tokyo, Japan; 8 Isotope Science Center, The University of Tokyo, Tokyo, Japan; 9 Showa Medical University College of Nursing, Tokyo, Japan

**Keywords:** tumor *Akkermansia muciniphila*, immunohistochemistry, PD-L1, immunecheckpoint inhibitors, biomarker

## Abstract

**Introduction:**

A prior retrospective analysis demonstrated that quantifying Programmed Cell Death Ligand 1 (PD-L1) expression using a phosphor-integrated dot (PID) score effectively predicted immune checkpoint inhibitor (ICI) efficacy in non-small-cell lung cancer (NSCLC) and other cancers. However, PD-L1 expression proved unreliable in some patients with low PD-L1 levels, highlighting the need for alternative biomarkers. A previous cohort study in NSCLC patients linked intestinal *Akkermansia muciniphila* (Akk) presence to improved ICI efficacy, particularly in low PD-L1 subgroups. Here, we evaluated tumor tissue Akk expression via immunohistochemical staining as a potential biomarker for ICI response in NSCLC.

**Methods:**

We retrospectively analyzed tumor tissues from 60 metastatic or recurrent NSCLC patients treated with ICIs. Immunohistochemical (IHC) staining was performed to assess Akk and PD-L1 expression, along with CD3 and CD68 in PD-L1-low samples. Transcriptomic profiling using RNA-sequencing was conducted on tumor samples to identify Akk-related gene expression patterns.

**Results:**

Tumor Akk expression showed no correlation with PD-L1 levels assessed via PID. Survival and multivariable Cox regression analyses revealed no association between Akk expression and progression-free survival (PFS) or overall survival (OS). In high PD-L1 patients, Akk status did not influence outcomes. However, among low PD-L1 patients, Akk-positive cases exhibited significantly worse PFS compared to Akk-negative cases (OS remained unchanged). Transcriptome analysis indicated that Akk positivity in low PD-L1 samples exhibited enrichment in oxidative phosphorylation and amyotrophic lateral sclerosis-related pathways and downregulation of spliceosome-associated pathways. No significant differences in tumor-infiltrating CD3+ T cells or CD68+ macrophages were observed between Akk-positive and Akk-negative tumors in the PD-L1-low group

**Conclusions:**

Tumor-associated Akk may serve as a negative predictive biomarker for ICI efficacy in NSCLC patients with low PD-L1 expression. Our findings suggest that tumor microbiota profiling, particularly targeting Akk, could refine patient stratification and therapeutic decision-making.

## 1 Introduction

In a previous retrospective analysis, we demonstrated that quantifying Programmed Cell Death Ligand 1 (PD-L1) expression using a phosphor-integrated dot (PID) score effectively predicted the efficacy of immune checkpoint inhibitors (ICIs) across various cancers, including non-small cell lung cancer (NSCLC) (1). However, PD-L1 expression was not a reliable predictor in some patients with low PD-L1 levels, highlighting the need for alternative biomarkers. Therefore, it is essential to elucidate molecular pathological mechanisms of ICI resistance beyond PD-L1.

*Akkermansia muciniphila* (Akk) plays multiple roles and has beneficial effects on systemic metabolism, immunity, the intestinal barrier, and tumor progression (2–4). A Study has shown that intestinal Akk is overrepresented in cancer patients with progression-free survival (PFS) of more than 3 months or in ICI responders (4). A recent cohort study also demonstrated that intestinal Akk was associated with ICI efficacy in NSCLC patients, particularly in those with low PD-L1 expression (2). Furthermore, in a lung cancer animal model, it was observed that intestinal Akk could enter the bloodstream and subsequently colonize lung cancer tissue (5).

Taken together, these findings led us to hypothesize that tumor Akk may be associated with prognosis or ICI efficacy of NSCLC patients, independent of PD-L1 expression.

## 2 Material and methods

### 2.1 Ethics statement

This study was conducted in accordance with the guidelines of the Declaration of Helsinki and was approved by the Ethics Committees of Showa University School of Medicine (approval number: 2772) and Fukushima Medical University (approval number: 2019-262). Informed consent was obtained from all patients involved in the study.

### 2.2 Patient selection

This study enrolled 60 NSCLC patients with metastatic or recurrent cancer who were treated with ICIs. It was a multicenter retrospective cohort study, and patients were diagnosed and treated at Showa University Hospital and Fukushima Medical University Hospital from December 2015 to December 2022. All patients received treatment regimens, including ICIs as shown in **Table 1**, which were administered according to clinical practice.

**Table 1:** Clinical, pathological and molecular characteristics of non-small lung cancer cases according to tumor *Akkermansia muciniphila* (Akk) expression.

Characteristic	*Akkermansia muciniphila*	
	Negative (N = 30)	Positive (N = 30)
Sex		
Male	27	22
Female	3	8
Mean age + SD (years)	68.5 + 9.1	68.5 + 8.7
Site of pathological specimen		
Primary tumor	25	25
Metastatic tumor	5	5
ICI Regimen		
Nivolumab monotherapy	13	21
Pembrolizumab monotherapy	12	8
Pembrolizumab + platinum-based chemotherapy	2	1
Atezolizumab + platinum-based chemotherapy	3	0
PD-L1 expression (PID score)		
Low (PID score<2000)	12	17
High (PID score>2000)	18	13
% Antibiotic use*	17	23
% Comorbidities*	68	70

*Eight weeks before and after ICI treatment. Comorbidities include diabetes, hypertension, dyslipidemia, hyperruricemia, cardiovascular diseases, COPD, abd absencesses. SD, standard deviation; ICI, immune checkpoint inhibitor; PID, phosphor-integrated dots.

### 2.3 Assessment of the treatment response

Each patient’s treatment response was evaluated using computed tomography scans for imaging assessments. Treatment efficacy was evaluated according to the Response Evaluation Criteria in Solid Tumors version 1.1 (6). Overall survival (OS) was defined as the time from the start of the first administration of treatment to the date of death from any cause or the last follow-up. Progression-free survival (PFS) was defined as the time from the start of treatment to the first documented instance of disease progression, death from any cause, or the last follow-up, whichever occurred first. The cut-off date for follow-up was set as December 2022.

The “median PFS” and “median OS” from phase III pivotal clinical trials were used to uniformly evaluate the treatment efficacy of patient populations. The patients were divided into two groups (responders and non-responders) based on their treatment response. We then performed an analysis to compare Akk and PD-L1 expression in each group.

### 2.4 Immunohistochemical analysis

All tumor tissue specimens used to evaluate Akk expression were obtained before each patient received ICI treatment. The immunohistochemistry (IHC) staining procedure using DAB, and the method of evaluating Akk expression, were performed according to standard clinical protocols. 60 Formalin-fixed, paraffin-embedded (FFPE) tissue samples obtained by biopsy or resection were prepared for analysis. FFPE cut to a thickness of 4 μm were deparaffinized and stained using an automated immuno-stainer BOND-III (Leica Biosystems, Germany) using the manufacturer’s IHC protocol. Antigen retrieval was performed using the BOND Enzyme Pretreatment Kit (Leica Biosystems, AR9551) at 37°C for 10 min, followed by incubation with *Akkermansia muciniphila* primary antibody (1:500, Sigma-Aldrich, SAB4200870, Germany) at room temperature for 15 min. DAB detection was performed using the BOND Polymer Refine Detection (Leica Biosystems, DS9800). Three independent pathologists independently evaluated all 60 immuno-stained slides. We defined Akk positivity as clear intracellular staining of Akk in multiple fields of view. Appropriate positive controls were included in each IHC run. The same tumor tissue specimens were used to evaluate PD-L1 expression. The method for PD-L1 PID scoring was conducted as previously reported (1).

We also performed immunofluorescence staining of CD3 and CD68 on specimens with low PD-L1 expression (n=30). We sliced 4-μm sections from paraffin blocks and placed them on glass slides, which were then deparaffinized and rehydrated. Antigen retrieval was performed by heating the fixed tissue sections in a Target Retrieval Solution High pH (Dako S3307; Agilent, CA, USA) at 98°C for 40 minutes. Tissue sections mounted on the glass slides were then blocked in Protein Block Serum-Free (Dako X0909; Agilent, CA, USA) for 5 minutes at room temperature and subsequently incubated with anti–CD3 mouse monoclonal antibody (NCL-L-CD3-565, clone LN10, 1:500; Leica Biosystems, Germany) and CD68 rabbit monoclonal antibody (#76437, clone D4B9C, 1:1000; Cell Signaling Technology, MA, USA) overnight at 4°C. After washing with phosphate-buffered saline solution, samples were stained with secondary antibodies (anti–mouse IgG–Alexa 488 and anti–rabbit IgG–Alexa 594, 1:200; Thermo Fisher Scientific, MA, USA) for 1 hour at room temperature. We counterstained cell nuclei with DAPI II COUNTERSTAIN (06J5001; Abott, IL, USA). An All-in-One Fluorescence Microscope (BZ-X810, KEYENCE, OSAKA, Japan) was used to assess the positivity according to double immunostaining, as previously reported (7).

### 2.5 RNA-sequencing library preparation

Total RNA from tumor tissues was isolated by RNeasy Micro kit (Qiagen, Netherlands). The RNA integrity score was calculated with the RNA 6000 Nano reagent (Agilent, CA, USA) in a 2100 Bioanalyzer (Agilent, CA, USA). RNA-Seq libraries were prepared with a The SMART-Seq^®^ Stranded Kit (# 634444, Takara Bio, Japan). The libraries were sequenced on the NovaSeq 6000 system (Illumina) as paired-end 150 base reads.

### 2.6 RNA- sequencing data analysis

RNA libraries were sequenced on an Illumina NovaSeq 6000 platform, generating 2 × 150 bp paired-end reads. Read alignment was performed using STAR (version 2.7.10a) with default parameters, mapping to the human genome (GRCh38) and transcriptome (GENCODE version 40) as reference datasets. Gene expression levels were quantified as fragments per kilobase of exon per million reads mapped (FPKM) using StringTie (version 2.2.1).

### 2.7 Scatter plot

To visualize the effects of Akk positivity on FPKM expression levels between Akk positive and Akk negative in all specimens (n=6 per group), PD-L1 low specimens (n=3 per group) and PD-L1 high specimens (n=3 per group).

### 2.8 KEGG pathway analysis

For RNA-sequencing data in all specimens, PD-L1 low specimens and PD-L1 high specimens, gene annotation enrichment analysis was performed for KEGG pathway analysis, using the functional annotation tool in DAVID Bioinformatics Resources 2021 (https://davidbioinformatics.nih.gov/home.jsp).

### 2.9 Statistical analysis

Statistical analyses were performed, and figures were generated using GraphPad Prism 8.4.3 software (GraphPad Software Inc., San Diego, CA, USA) or JMP software (SAS institute., NC, USA). Spearman’s correlation coefficient was used to analyze the associations between variables. Un-paired t test was used to compare values between two groups. Statistical significance was defined as a p-value <0.05.

For survival analyses, survival durations (PFS and OS) were assessed using the Kaplan–Meier method and Cox proportional hazard model. All tests were two-sided. When comparing two groups using the log-rank test, p-values <0.05 were considered statistically significant.

## 3 Results

### 3.1 Clinicopathological characteristics

**Figure 1:**
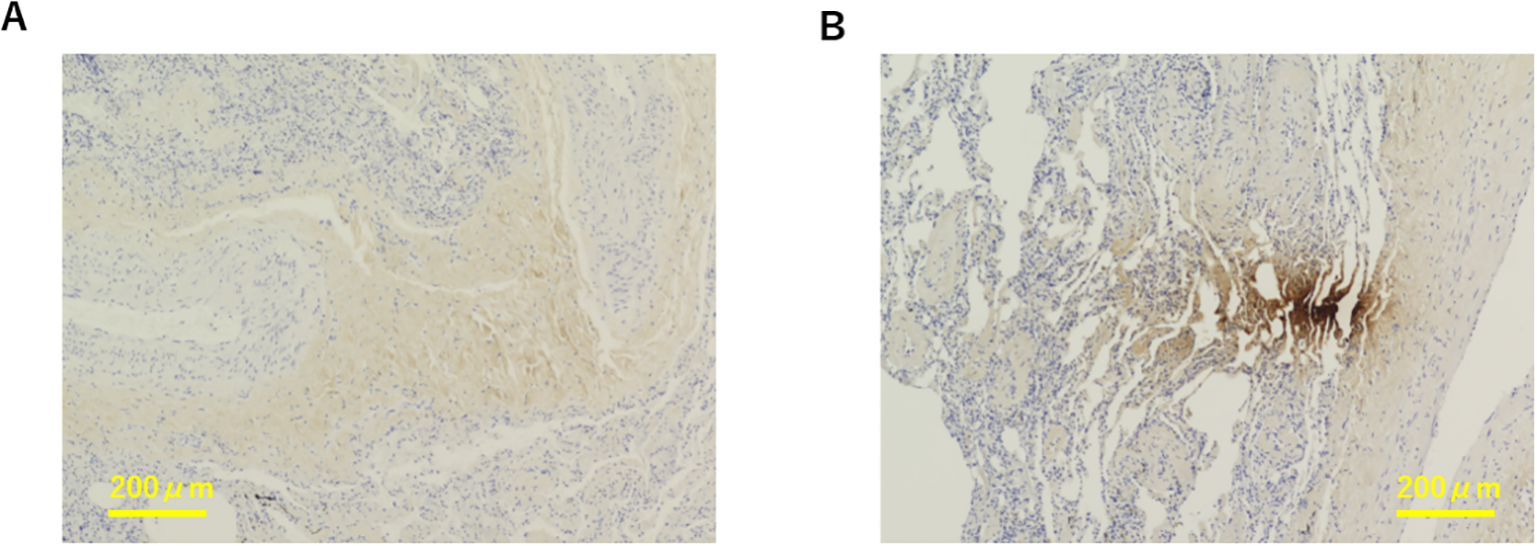
Representative images of Akk-negative **(A)** and Akk-positive tumors **(B)**. The yellow bar represents 200 µm.

Representative images of Akk-negative and Akk-positive tumors are shown in **Figure 1**. The clinicopathological characteristics of the 60 NSCLC patients with available tumor Akk expression data are summarized in **Table 1**. As previously reported, PD-L1 expression was categorized as low or high, with a PID score of <2000 or >2000, respectively (1). The majority of patients received ICI monotherapy (Nivolumab or Pembrolizumab), with 83.3%(25/30) of Akk-negative and 96.7%(29/30) of Akk-positive patients undergoing this treatment. Potential confounding factors, such as antibiotic use or comorbidities, were not different between them.

### 3.2 Correlation between tumor Akk and PD-L1 expression

**Figure 2:**
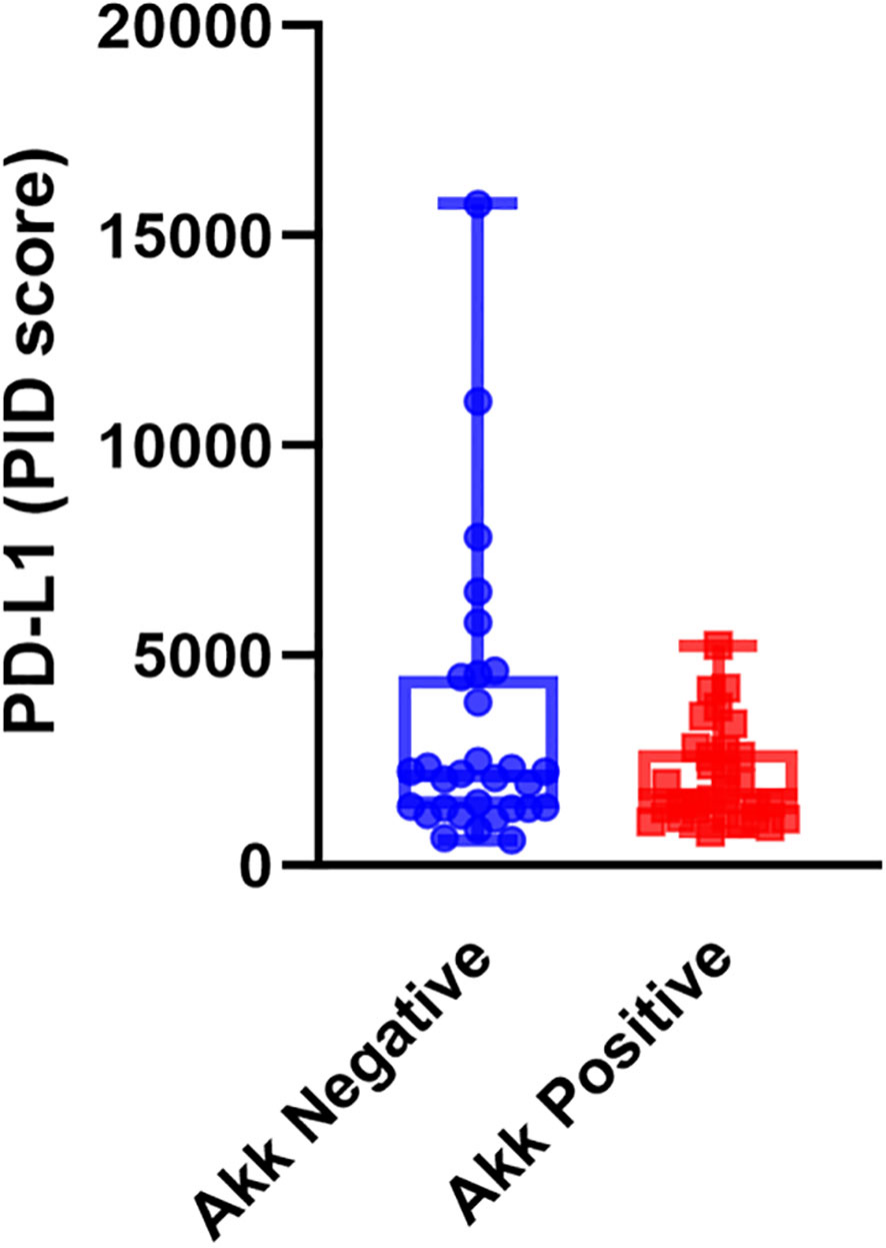
PD-L1 expression assessed by the PID method in Akk-negative (n=30) and Akk-positive tumors (n=30). Tumor Akk expression was not significantly associated with the PD-L1 PID score (*P=0.082*, unpaired t-test).

We examined the correlation between tumor Akk and PD-L1 expression using the PID method. Tumor Akk positivity was not significantly associated with the PD-L1 PID score (**Figure 2**, *P=0.082*, unpaired t-test).

### 3.3 Kaplan–Meier survival analysis according to tumor Akk and PD-L1 expression

**Figure 3:**
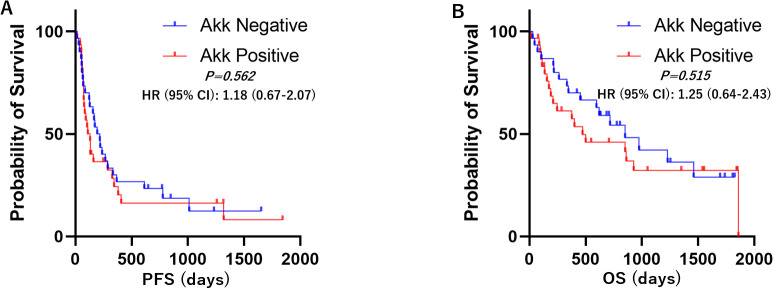
Kaplan–Meier survival analysis according to tumor Akk expression. There was no significant difference in **(A)** PFS or **(B)** OS between Akk-negative (n=30) and Akk-positive tumors (n=30). HR, hazard ratio; CI, confidence interval.

**Figure 4:**
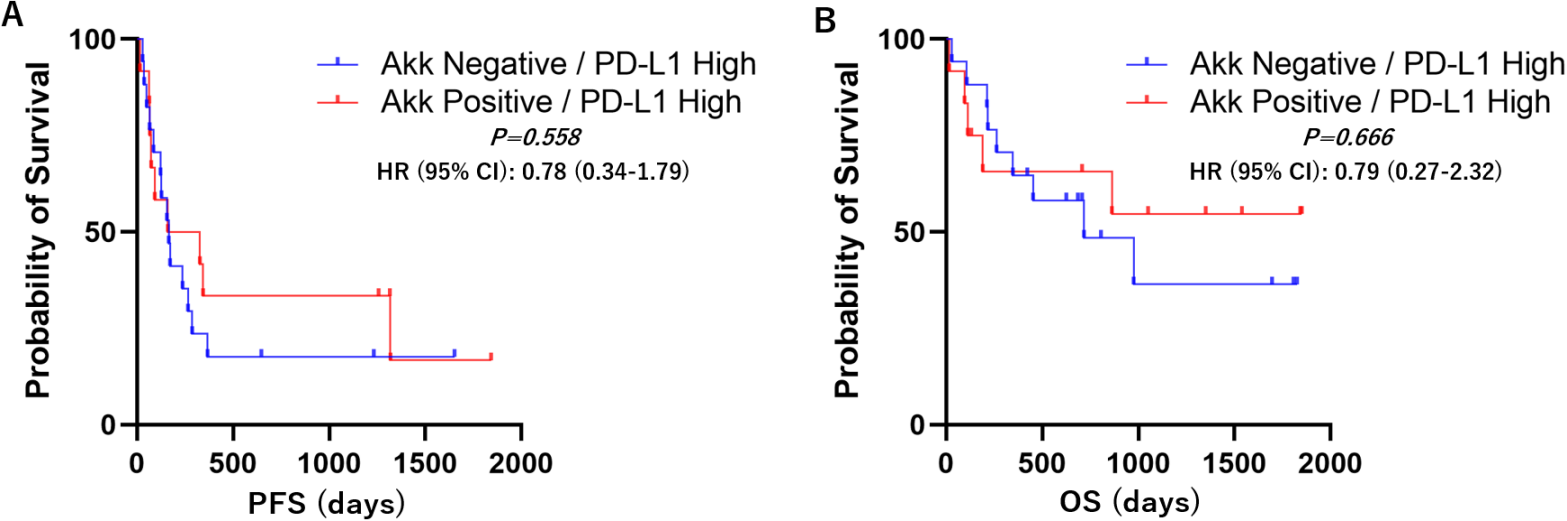
PD-L1 high; Kaplan–Meier survival analysis according to tumor Akk expression. There was no significant difference in **(A)** PFS or **(B)** OS between Akk-negative (n=18) and Akk-positive tumors (n=13). HR, hazard ratio; CI, confidence interval.

**Figure 5:**
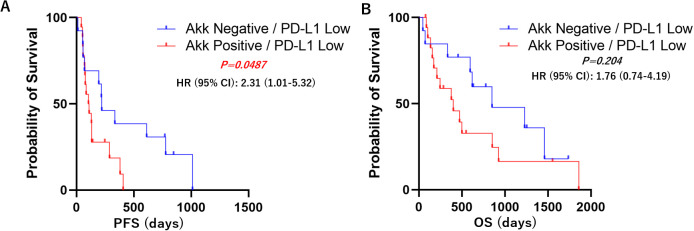
PD-L1 low; Kaplan–Meier survival analysis according to tumor Akk expression. Akk-positive patients (n=17) had worse **(A)** PFS compared to Akk-negative patients (n=12), although there was no significant difference in **(B)** OS. HR, hazard ratio; CI, confidence interval.

Kaplan–Meier survival analyses with log-rank tests were performed to compare progression-free survival (PFS) and overall survival (OS) between the Akk-negative and Akk-positive groups. In the overall patient population (n=60), there was no significant difference in PFS or OS between the two groups (**Figure 3**, Hazard ratio = 1.18 (0.67-2.07) or 1.25 (0.64-2.43)). Similarly, in patients with high PD-L1 expression, Akk status was not associated with either PFS or OS (**Figure 4**, Hazard ratio = 0.78 (0.34-1.79) or 0.79 (0.27-2.32)). However, among patients with low PD-L1 expression, Akk-positive patients had worse PFS compared to Akk-negative patients (**Figure 5**, Hazard ratio = 2.31 (1.01-5.32), *P = 0.0487*), although there was no significant difference in OS [Hazard ratio = 1.76 (0.74-4.19)].

Univariable or multivariable Cox regression analysis was performed to adjust for potential confounders (sex, age, PD-L1 expression, or ICI regimen). There was no significant difference in PFS or OS between the two groups (**Table 2**).

**Table 2:** The exisetnce of *Akkermansia muciniphila* in non-small lung cancer (NSCLC) tissue and patient mortality.

Characteristic	NSCLC specific mortality			Overall mortality		
	No. of events	Univariable	Multivariable	No. of events	Univariable	Multivariable
		HR (95% CI)	HR (95% CI)*		HR (95% CI)	HR (95% CI)*
Akkermansia muciniphila						
Negative (N=30)	25	1 (reference)	1 (reference)	17	1 (reference)	1 (reference)
Positive (N=30)	25	1.18 (0.67 to 2.06)	1.05 (0.57 to 1.94)	19	1.25 (0.64 to 2.43)	1.21 (0.62 o 2.36)

*The multivariable Cox regression model initially included sex, age, PD-L1 expression,a nd ICI regimen for NSCLC specific mortality, or sex, age, and PD-L1 expression for overall mortality.

In summary, these results suggest that tumor Akk expression may serve as a predictive marker for ICI efficacy in NSCLC patients with low PD-L1 expression, but not in those with high PD-L1 expression.

### 3.4 Transcriptomic analysis according to tumor Akk positivity

**Figure 6:**
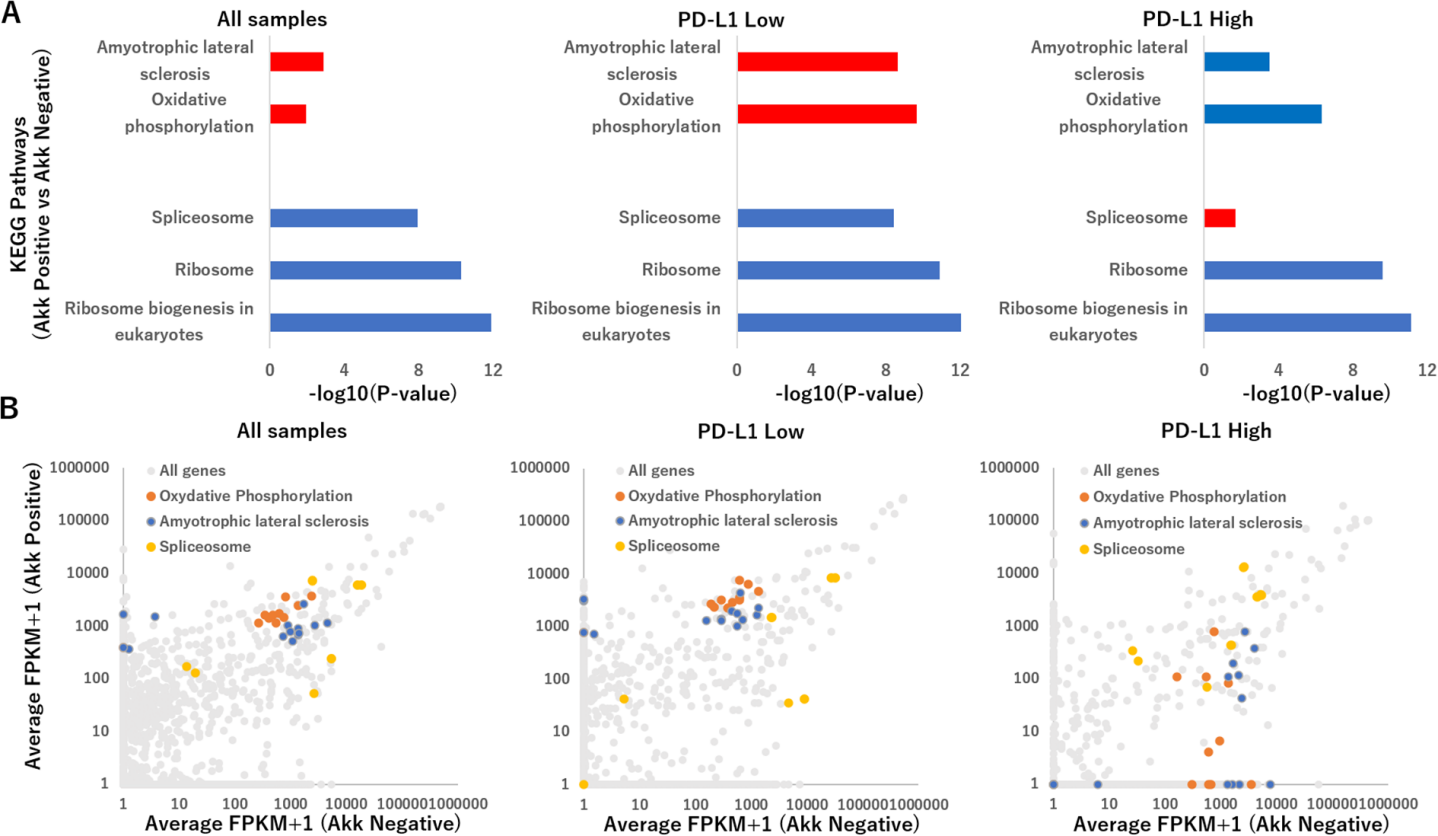
**(A)** Summary of the Kyoto Encyclopedia of Genes and Genomes (KEGG) pathway analysis for top 100 upregulated or downregulated genes tumor Akk positivity effected the gene expressions in all specimens (n=6 per group, left), PD-L1 low ones (n=3 per group, middle) and PD-L1 high ones (n=3 per group, right), using the Functional Annotation tool at DAVID Bioinformatics Resources. The representative pathways were expressed, using −log10(P-value), which was calculated via a Fisher’s exact value. Upregulated and downregulated pathways were shown with red and blue bars. **(B)** Scatterplot of genes altered in Akk positive versus Akk negative in all specimens (left), PD-L1 low ones (middle) and PD-L1 high ones (right), depicting average FPKM values. Genes related to amyotrophic lateral sclerosis, oxidative phosphorylation and spliceosome were shown with orange, blue, yellow colors.

RNA-sequencing was performed to understand how tumor Akk positivity effected the gene expressions in all specimens (n=6 per group), PD-L1 low ones (n=3 per group) and PD-L1 high ones (n=3 per group). All RNA-sequencing results are provided in **Supplementary Table S1**. A total of 7,854 genes were expressed in one of the replicates in all groups. In Akk positive samples, compared to Akk negative samples, the groups of genes showing the top 100 enhanced or suppressed expression were defined as upregulated or downregulated genes, respectively. For these genes, Kyoto Encyclopedia of Genes and Genomes (KEGG) enrichment analysis was performed using the Database for Annotation, Visualization, and Integrated Discovery (DAVID software). In all samples or PD-L1 low ones, Akk positivity upregulated pathways linked to amyotrophic lateral sclerosis and oxidative phosphorylation, while downregulating pathways linked to Ribosome biogenesis in eukaryotes, Ribosome, and spliceosome (**Figure 6A**). On the other hand, in PD-L1 low ones, Akk positivity suppressed these pathways other than a spliceosome pathway (**Supplementary Table S2**).

**Figure 6B** displayed RNA sequencing results as a scatter plot for two-group comparisons: Akk positive versus Akk negative in all specimens, PD-L1 low ones and PD-L1 high ones. In all samples or PD-L1 low ones, Akk positivity upregulated genes related to amyotrophic lateral sclerosis (such as PSMB5, PFN1, SRSF3, NDUFS5, MT-CO2, MT-CYB, MT-ND2, MT-ND5, MT-CO1, MT-ND4, MT-ND1, MT-CO3, MT-ND4L, MT-ATP8) and oxidative phosphorylation (NDUFS5, MT-CO2, MT-CYB, MT-ND2, MT-ND5, MT-CO1, MT-ND4, MT-ND1, MT-CO3, MT-ND4L, MT-ATP8), while downregulating pathways linked to Ribosome biogenesis in eukaryotes, Ribosome, and spliceosome (HNRNPC, DDX5, RNVU1-7, RNU1-1, RNVU1-18, RNU1-2, RNU6-2, RNU1-4, RNU6-9, RNU1-3, LOC124904613). On the other hand, in PD-L1 high ones, Akk positivity suppressed genes related to these pathways other than spliceosome.

### 3.5 Tumor-infiltrating immune cells according to tumor Akk in PD-L1 low specimens

**Figure 7:**
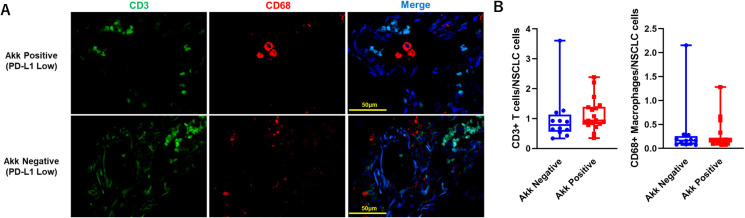
**(A)** Representative double immunostaining for CD3 and CD68 in specimens with PD-L1 low expression (n=30). **(B)** The number of CD3+ T cells or CD68+ macrophages between Akk-negative (n=12) and Akk-positive tumors (n=18) were counted. Theses expressions were not different (*P=0.64* or *0.76* for CD3+ T cells or CD68+ macrophages, unpaired t-test).

Because Akk induces homeostatic immune responses (3), we evaluated Tumor-Infiltrating Immune Cells, such as CD3+ T cells or CD68+ macrophages, in specimens with PD-L1 low expression (**Figure 7A**, n=30). The number of CD3+ T cells or CD68+ macrophages between Akk-negative (n=12) and Akk-positive tumors (n=18) were not different (**Figure 7B**, *P=0.64* or *0.76*, unpaired t-test).

## 4 Discussion

Here, we report the results of a retrospective, multicenter study on NSCLC patients treated with ICIs. Tumor Akk expression was associated with poor prognosis or non-response to ICIs in patients with low PD-L1 expression, but not in those with high PD-L1 expression. This result contrasts with findings regarding intestinal Akk, where ICI efficacy and prognosis were better in patients with a low abundance of intestinal Akk compared to those with high abundance or absence. This tendency was particularly observed in patients with low PD-L1 expression, but not in those with high PD-L1 expression (2). Recently, in a lung cancer animal model, it has been shown that intestinal Akk can enter the bloodstream and subsequently colonize lung cancer tissue (5). It is possible that the effect of tumor Akk on cancer immune response differs from that of intestinal Akk. Recent studies have highlighted the complexity of the tumor microbiome and its impact on cancer therapy. Nejman et al. demonstrated that various tumor types harbor distinct intracellular bacteria, which can modulate immune responses and influence treatment outcomes (8). In addition to Akk, other genera such as *Bifidobacterium* and *Ruminococcus* have been implicated in modulating ICI efficacy (9). The functional consequences of these bacteria within the tumor microenvironment remain to be fully elucidated.

Within the tumor microenvironment, Akk may interact with immune cells, modulate metabolic pathways, or influence the local immune milieu. Our transcriptomic analysis revealed that Akk positivity in PD-L1-low tumors was associated with upregulation of oxidative phosphorylation and neurodegenerative disease pathways, and downregulation of spliceosome-related pathways, suggesting a potential impact on tumor cell metabolism and immune regulation. Low intestinal Akk expression correlates with increased oxidative stress and inflammatory responses (10). Although multiple models have shown that Akk in intestinal bacteria may contribute to the pathogenesis of ALS via oxidative stress alleviation and activation of the PI3K/Akt pathway (11, 12), there is currently no clear evidence regarding its association with the spliceosome. Alternative splicing of PD-1 or PD-L1 induces the production of soluble PD-1 or PD-L1, which suppresses the tumor immune response (13). However, it remains unclear how alternative splicing affects other immune checkpoints. Although this is the first report of transcriptome analysis of tumor Akk, it is necessary to elucidate the interaction between Akk and tumor microenvironment constituent cells and its relationship with spliceosomes in the future.

Recent findings indicate that the majority of intratumoral microbiota are encapsulated within tumor-infiltrating macrophages (14). It has also shown that Akk secretes threonyl-tRNA synthetase (AmTARS) riggers M2 macrophage polarization and orchestrates the production of anti-inflammatory IL-10 (15). Furthermore, *in vitro* studies on the interaction between Akk and macrophages revealed that repeated exposure to Akk induced the upregulation of certain immune checkpoints, such as the Siglec family, but not PD-L1 (16). This resulted in increased bacterial intracellular survival and reduced inflammation. In this study, these immune checkpoints could not be detected well by RNA-sequencing. Therefore, Akk derived proteins or metabolites could enhance the expressions of immune checkpoint factors other than PD-L1. Further molecular studies are needed to elucidate this pathogenesis.

This is the first study to detect proteins derived from tumor microbes of NSCLC patients, using a anti Akk antibody. Until now, methods for studying tumor microbes have focused on targeting DNA or RNA (14). However, detecting DNA or RNA in FFPE samples can sometimes be challenging compared to detecting proteins. This new method could improve our understanding of the tumor microbiome in various cancers.

The limitations of this study are as follows: First, it was a retrospective analysis and primarily exploratory, examining the utility of tumor Akk expression through immunohistochemistry (IHC). Second, unlike previous studies on tumor microbiota that employed RNA *in situ* analysis (10), we did not conduct such an analysis here. Third, interactions between tumor mutational burden and tumor Akk expression were not considered. Fourth, RNA sequencing results varied widely because the quality of FFPE-derived RNA varied widely from sample to sample. Additionally, this study did not investigate the functional and clinical relevance of Akk commensals, such as *Bifidobacterium adolescentis* (2).

Nevertheless, our results suggest that tumor microbiota profiling, particularly targeting Akk, may provide additional prognostic information, especially for NSCLC patients with low PD-L1 expression. Combining tumor microbiome analysis with established biomarkers such as PD-L1, tumor mutation burden, and immune cell infiltration could improve patient stratification and guide therapeutic decision-making. Further studies are warranted to validate these findings and explore the therapeutic potential of modulating the tumor microbiome.

## 5 Conclusions

In conclusion, we propose that tumor Akk abundance could serve as a reliable biomarker for poor prognosis in low PD-L1 expression NSCLC patients receiving ICIs.

## Data Availability

The original contributions presented in the study are included in the article/**Supplementary Material**, further inquiries can be directed to the corresponding author/s.
