# Correction: Tumor *Akkermansia muciniphila* predicts clinical response to immune checkpoint inhibitors in non-small-cell lung cancer patients with low PD-L1 expression

**DOI:** 10.3389/fimmu.2026.1827736

**Published:** 2026-03-26

**Authors:** Takashi Shimizu, Ryotaro Ohkuma, Mayumi Homma, Shingo Nakayama, Yosuke Sasaki, Satoshi Muto, Katsuaki Ieguchi, Makoto Watanabe, Akashi Taguchi, Daisuke Takayanagi, Youichiro Wada, Atsushi Horiike, Yutaro Kubota, Hirotsugu Ariizumi, Masahiro Shimokawa, Yuya Hirasawa, Tomoyuki Ishiguro, Risako Suzuki, Nana Iriguchi, Emiko Mura, Kiyoshi Yoshimura, Mayumi Tsuji, Yuji Kiuchi, Hiroyuki Suzuki, Toshiko Yamochi

**Affiliations:** 1 Department of Clinical Diagnostic Oncology, Clinical Research Institute for Clinical Pharmacology and Therapeutics, Showa Medical University, Tokyo, Japan; 2 Clinical Research Institute for Clinical Pharmacology and Therapeutics, Showa Medical University, Tokyo, Japan; 3 Division of Medical Oncology, Department of Medicine, School of Medicine, Showa Medical University, Tokyo, Japan; 4 Department of Pathology, Showa Medical University School of Medicine, Tokyo, Japan; 5 Department of Chest Surgery, School of Medicine, Fukushima Medical University, Fukushima, Japan; 6 Department of Pharmacology, School of Medicine, Showa Medical University, Tokyo, Japan; 7 Pharmacological Research Center, Showa Medical University, Tokyo, Japan; 8 Isotope Science Center, The University of Tokyo, Tokyo, Japan; 9 Showa Medical University College of Nursing, Tokyo, Japan

**Keywords:** tumor *Akkermansia muciniphila*, immunohistochemistry, PD-L1, immunecheckpoint inhibitors, biomarker

In the acknowledgements, the following information was erroneously omitted: Takuya Tsunoda, Shinichi Kobayashi, and Satoshi Wada contributed to the collection of clinical data from patients, validated the reproducibility of the data analysis, and provided direction and supervision for the project.

The original version of this article has been updated.

